# A sustainability framework based on threats, consequences, and solutions (TCS) for managing watershed commons

**DOI:** 10.1371/journal.pone.0295228

**Published:** 2023-12-06

**Authors:** Ana Lorena Quiñónez Camarillo, Timothy O. Randhir

**Affiliations:** Department of Environmental Conservation, University of Massachusetts, Amherst, Massachusetts, United States of America; Feroze Gandhi Degree College, INDIA

## Abstract

Sustainable management of common pool resources requires local information and participation. We develop a framework for managing commons based on threats, consequences, and solutions (TCS). The status of the community’s interaction with their local commons is critical in developing viable solutions to avoiding the loss of natural resources, enhancing the benefits they provide, and sustaining the functions they perform. Threats to natural resources, the consequences of their depletion, and the solutions local communities perceive as most effective to prevent this loss are assessed as related to socioeconomic and landscape factors to develop strategies for the resilience of commons. Communities and representative stakeholders (224 respondents) participated in a survey in Honduras’s Lake Yojoa watershed. The community’s perception was also evaluated for impacts of changes in land use and climate on local commons. An ordinal logistic regression analysis was used to determine the effect of land use, geographic, and demographic factors on community perceptions. Distance to the lake, landcover percentages, slope, type of work, age, and importance of tourism were significant in influencing community interaction and perception of TCS. The involvement of communities in deriving knowledge on TCS is critical to increasing the resilience of local commons to emerging threats.

## Introduction

### Loss of local commons

Local communities worldwide, both in rural and urban areas, depend on common pool resources [1, 2] to sustain their livelihoods, especially in developing countries [3]. Common pool resources are depletable and non-excludable, challenging their management [4]. Increasing demand for resources drives the overexploitation of these resources and decreases their capacity to provide critical ecosystem services and comply with the ecosystem functions they perform [5, 6]. For example, since 2000, tropical rainforests have experienced approximately 6.5 times more deforestation than since 1990 [7]. Central and South America have the highest percentage of decline in amphibian species, while Indonesia, India, and Brazil are among the countries that have the most threatened mammals and bird species [8]. Losing ecosystem services and functions can severely affect our livelihood, health, and survival [9, 10]. Therefore, it is crucial to understand how ecosystem stakeholders interact with their local commons to suggest viable solutions to the increasing loss of natural resources, the benefits they provide, and their functions. To do this, we have developed a community-based framework for analyzing threats, consequences, and solutions (TCS) that will allow us to gain insight into stakeholder knowledge and their interactions with resources.

### Need for resilience

Globalization impacts [11], land use change [12], and climate change [13, 14] can accelerate the degradation of common pool resources and enhance community vulnerability to these changes. For example, a decline in biodiversity and forest product availability can result from large-scale forest clearings, resulting in decreased productivity, biodiversity loss, and enhanced drying of the forest floors. As we lose our healthy forests, we also lose ecosystem services such as carbon storage, water balance, river flow regulation, ameliorating infectious diseases, and regulation of regional climate patterns [15]. In addition, biodiversity loss affects ecosystem processes that provide ecosystem services such as food, potable water, shelter, and medicines [16, 17]. These processes include soil formation and retention, plant biomass production, nutrients, and water cycling [16]. River degradation affects critical ecosystem services such as the provision of water and energy, fisheries, temperature regulation, erosion, and flood control [18]. For example, dam construction in rivers affects fisheries and local sustainability [19]. According to the Millennium Ecosystem Assessment [10], over 60% of the assessed ecosystem services are declining due to anthropogenic activity and exploitation. Therefore, there is a need to study local knowledge of threats, the consequences of the threat, and perceived solutions to improve community/stakeholder resilience to multiscale-level disturbances [13, 20, 21].

### Role of local knowledge in resilience

Resilience is defined as the capacity of a system to absorb disturbance and reorganize to retain the same function, structure, feedback, and identity [22, 23]. Traditional ecological and local knowledge is vital for socioecological resilience [24, 25]. Local knowledge is a primary resource for understanding vulnerability and increasing resiliency at the community level [26–30]. Not all stakeholders will interact with and depend on their common pool resources in the same way; therefore, their adaptation to the loss of ecosystem services (resilience) can also vary. Values, attitudes, and perceptions are defining factors in how people relate to the environment, conservation issues, and environmental decision-making processes [31–33]. Therefore, it is crucial to consider how humans interact with the environment and the factors that drive these interactions [34]. Ecosystem management requires a balance between the competing priorities and needs of the stakeholders involved, which, unfortunately, may agree in some areas but not others [35, 36]. Conflict arises when ecosystem management efforts cannot balance stakeholder needs, and conservation efforts impact human livelihood [37–39]. The role of local knowledge in managing commons is critical to sustainable outcomes [1].

### The TCS framework

**Fig 1:**
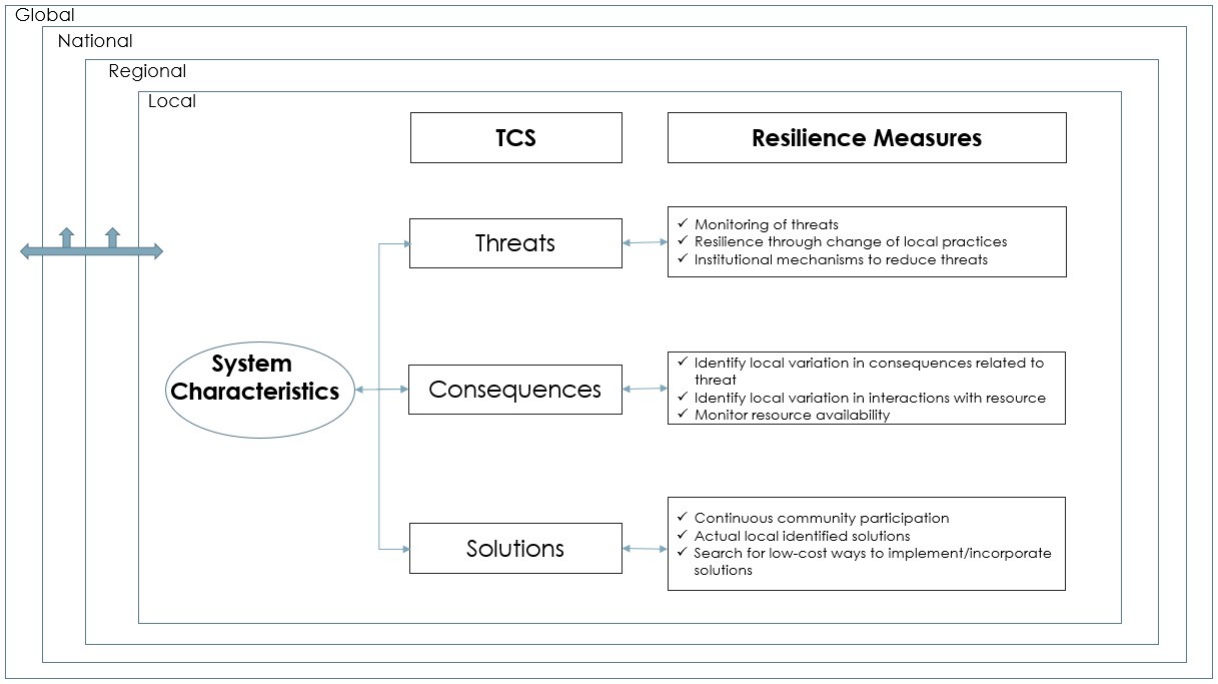
TCS framework based on the multiscale ecosystem framework [21].

A comprehensive multiscale framework is developed in this study to examine the local knowledge of TCS towards improving community resilience to multiscale disturbances. The TCS framework (Fig 1) is developed based on a multiscale ecological framework (MEF) [11, 21]. This study was designed to understand the underlying social context that drives stakeholder-resource interactions and could affect the threats to natural resources, the consequences of the loss of these resources, and the solutions that the communities perceive will be the most effective options to reduce the threats to these resources. It is necessary to include stakeholder participation in planning for ecosystem resilience [28–30]. Heterogeneity in cultural, economic, social, and political factors is influential in developing effective management strategies [39–41]. By understanding and integrating the stakeholders’ interests, management strategies developed from considering various factors can be more holistic and sustainable[42, 43]. Therefore, it is crucial for resource conservation efforts to evaluate drivers of the human-ecosystem relationship, merge the underlying social context with the material impacts, and assess alternative approaches that will provide more adequate solutions [43–45]. Socioecological systems (SES) are analyzed as adaptive systems that combine social and environmental factors [22].

This study develops a unique TCS framework for analyzing local commons using watershed-based assessments. Studies on ecosystems and their degradation need assessment of system-wide interactions and processes. This approach is particularly relevant in many tropical countries, where economic resources and information availability are critical for research. For example, watershed-wide assessments are used to study degradation in Puerto Rico and Brazil [46–49], Malaysia [50, 51], and Thailand [52]. Despite recognizing the need for watershed-wide assessment, resource managers in tropical countries sometimes do not explicitly consider social and economic drivers when making ecological decisions, leading to conflict and poor outcomes [32, 37, 53]. However, there is a need for broader systems-based analysis using local information on ecosystem services and socioecological systems. To manage ecosystems and understand the effect of anthropogenic activities, studies on the whole system also need to include biotic and abiotic components and their interactions in watershed systems in their assessment [46, 54, 55]. To address these needs, this study explores the local context that drives the perceptions and values of watershed stakeholders regarding several common pool resources in Lake Yojoa.

### Objectives

1. to develop a conceptual framework based on TCS (treats, consequences, solutions) to assess factors driving stakeholder-resource interactions in local commons.
2. to evaluate social and ecosystem characteristics influencing the resilience of local commons and
3. To evaluate the perceptions toward TCS components among local communities in Lake Yojoa.

## Methodology

### Study area

**Fig 2:**
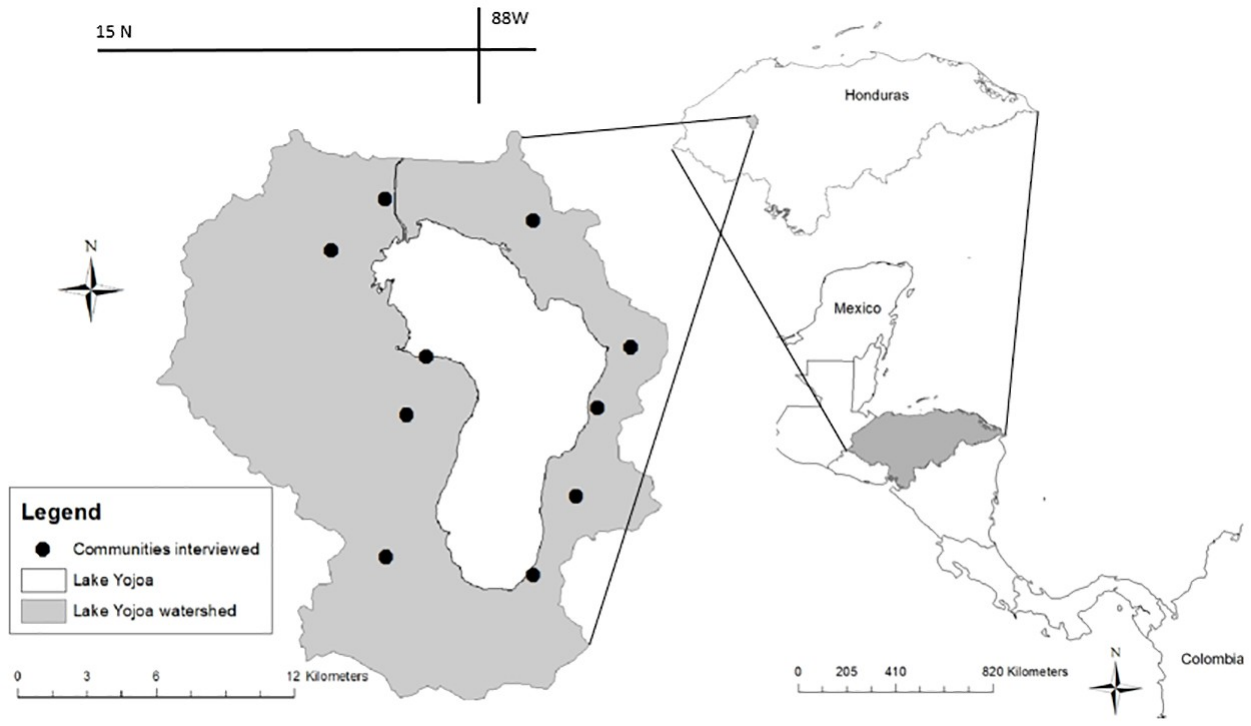
The map depicts the location of the communities interviewed for our survey. Maps are created by authors in GIS using base maps provided by the office of the Honduras Institute of Forest Conservation (ICF, http://geoportal.icf.gob.hn/geoportal/main).

The Lake Yojoa watershed is in the middle area of Honduras (Fig 2) and intersects the departments of Santa Bárbara, Cortés, and Comayagua. The watershed covers 337 km^2^, and Lake Yojoa is the largest body of water with an area of 83.5 km^2^ and a maximum depth of 29m. National Congress classified the Lake Yojoa watershed as a Reserve of Multiple Use (1971), which means its resources are to be used in a regulated manner to maintain the area’s ecological balance.

The Lake Yojoa watershed is considered an important resource in Honduras. First, it encompasses the country’s largest natural lake, and the watershed contains sections of two national parks that still have three classes of virgin forests. Secondly, it is an important natural area for the Mesoamerican Biological Corridor (MBC), home to many natural ecosystems and cultural heritage. Finally, it supports artisanal fisheries with an annual value estimated at least US$207,000 and probably up to US$345,000. In 2002, 53 communities lived in the Lake Yojoa watershed, with 74,624 inhabitants.

On the local level, two national parks surround the basin, and the organizations responsible for them are considered stakeholders. They are the *Fundación Ecológica Parque Nacional Montaña de Santa Bárbara* (Ecological Foundation National Park Santa Barbara Mountain, Fecomol) and *Parque Nacional Azul Meambar* (National Park Azul Meambar, Panacam). The *Asociación de municipios del Lago de Yojoa y su área de influencia* (Association of Municipalities of Lake Yojoa and its area of Influence, AMUPROLAGO) is a local collaboration whose purpose is to promote the conservation of the Yojoa basin, through the development of projects, research, and collaboration. Other stakeholders that need to be included for the effectiveness of suggested policies are the communities that live in the watershed, farmers and ranchers, fishermen, businesses such as hotels and restaurants, *Minas el Mochito*, and Aquafinca Saint Peter Fish.

On the west side of the watershed, the topography is irregular near the shore and becomes mountainous and steep when moving away from it. On the eastern side, a succession of hills, valleys, ravines, and cliffs can be observed until reaching a maximum elevation of 2,047m above sea level [56]. The lake’s climate is tropical in transition to subtropical, with a dry season (December to May) and a rainy season (June to December). The northern region of the lake receives the most precipitation (above 3000 mm), which decreases when moving toward the south end (1,600mm). The area’s average temperature varies between 21–24 C° [56]. Several factors have been recognized to affect the health and sustainability of the area. These include inadequate aquaculture and agriculture practices, infrastructure development, natural resources extraction, inadequate livestock management, mining, deforestation, hunting, contamination due to the use of agro-toxic products and lack of water treatment plants, invasive species introduction, dams to produce electric energy, change of natural flow direction and wetland removal [57, 58].

The variable topography of the watershed reflects the nature of economic opportunities. Jobs in the region vary a lot, and many times are variable by location. Areas higher in the mountain and more challenging to reach tend to focus more on agriculture and small businesses. Areas closer to the lake and the main roads have a greater variety of jobs related to fishing, tourism (restaurants, stores, guides, hotels, clerks, drivers), mines, tilapia or chicken farms, energy production, agriculture, and other local businesses. Other jobs observed were construction, carpentry, mechanics, and teaching. It is also common for women to stay at home and be homemakers. This information on spatial variability in opportunities helps assess TCS.

Our research also allowed us to identify several important stakeholders to consider in conservation efforts. Several stakeholders were associated with the Yojoa watershed conservation. On the governmental level, several institutions are involved, including the *Instituto de Conservación Forestal*, *Areas Protegidas y Vida Silvestre* (the Institute for the Conservation of Forests, Protected Areas and Wildlife, ICF), *Secretaría Nacional de Recursos Naturales y Ambiente* (National Secretary of Natural Resources and the Environment, SERNA), Dirección General de Biodiversidad (General Office for Biodiversity, DiBio), *Dirección General de la Pesca* (General Office of Fisheries, DIGEPESCA). These institutions are all responsible for conserving the area’s natural resources in diverse ways. In addition, the *Empresa Nacional de Energía Eléctrica* (National Company for Electric Energy, ENEE) is responsible for the dam in the lake and the other dams on its tributaries.

### Conceptual model

The Multiscale Ecosystem Framework [11, 21] uses a nested framework that considers system-wide changes; it includes the economic, ecological, and social systems of various commons and can guide the evaluation of inter-scale interactions. The TCS framework (Fig 2) derives from the Multiscale Ecosystem Framework and is used as a conceptual model to guide this research. The TCS framework simplifies the local information into three components (perception of threats, consequences, and solutions) to make the assessment of complex information easily understandable and relatable by the respondents in evaluating local commons. The nature of threats, consequences, and solutions involve potential implications that cross multiple spatial scales (individual, community, and regional). The TCS framework considers three main components: threats to, consequences of loss, and solutions to the loss of specific system characteristics. Each system characteristic is analyzed individually to perceive the variation in conflicts and interactions between the populations and the resource of interest.

### Survey

A survey was developed to assess the perception of the value of the local ecosystems and their services. Land use scenarios (threats) were presented to elucidate a qualitative response from the interviewees, focusing on the threats to the resources, the impacts of these scenarios on their lives, and the solutions they considered ideal for preventing or solving them. A total of 224 surveys were conducted. Respondents were members of 12 local communities in the lake’s watershed (Fig 2). Four communities were grouped as pairs, as the distance between them was negligible. The population size for the selected communities varied between 109 and 1,490 individuals. Twenty members of each community were chosen to answer this survey. The sampling aim is to cover the whole community, and an equal number of surveys per community was used rather than a proportion based on community size. The equal sample size was appropriate for the objectives of this study and allowed broader coverage of resource conditions of communities spread throughout the watershed compared to a proportional sample size. A combination of random selection and snowballing techniques [59] was applied. People were initially chosen randomly as possible interviewees as the surveyor walked through the community. The snowball technique was used by asking at the end of the survey (if the person decides to participate) or after the closing statement (if the person chooses not to participate) if they knew anybody in their community interested in participating. If no other person was suggested, the surveyor continued moving around the community and would choose another person randomly. The snowballing technique is criticized as it relies on existing networks and can introduce bias into research. First, there is a specific loss of control over how the sample is made. There is a higher possibility that interviewees could direct the interview toward other individuals with similar beliefs or characteristics, and the interviewer cannot guarantee representation. Nevertheless, we minimized this potential bias by including random selection when there is such potential bias. The snowball technique is helpful in this research to reach inaccessible groups [60] and tap into respondents’ unique social knowledge [61].

Twenty-four other stakeholders (private companies, governmental institutions, protected areas, etc.) were also interviewed since their input and participation can be crucial for successfully implementing conservation initiatives. These organizations were chosen because of their direct involvement with the communities and upon suggestion from conversations with AMUPROLAGO. AMUPROLAGO is the local commonwealth for the lake watershed that is extensively involved in the conservation efforts for this region. The survey was reviewed and approved by the IRB of the University of Massachusetts (IRB: #914 2019–5491). All participants were explained about the study, and oral informed consent was obtained and documented from each participant before proceeding with the survey. A verbal consent was obtained from each participant. An IRB-approved study information sheet was given to participants. Only individuals who agreed to consent were allowed to participate in the survey. Oral consent was documented by making a mark in the survey sheet of those individuals who agreed to participate, and no personal information was recorded. A member of AMUPROLAGO witnessed the survey since they accompanied the surveyors during all visits for logistic purposes.

Each of the towns was visited, and subjects were interviewed in person. The surveys were implemented orally and in Spanish. The interviewees’ answers were entered into the questionnaire. The respondents from each community were chosen through a combination of random selection and snowballing; therefore, age ranges, gender, ethnic background, and type of subjects were varied. Survey exclusions included children (0–17 yrs.) and individuals who expressed limitations because these individuals might not have the decision capacity to provide voluntary informed consent and are therefore considered potentially vulnerable.

### Data and analysis

The survey included four questions to obtain demographic data from the interviewees: community, estimated age, time living in the area, and job (Table 1). It also had a question to assess the importance of tourism in the area. Lastly, the rest of the survey included three questions divided into three components: threats, consequences of loss, and solutions to the loss. For each natural resource (forests, wetlands, wildlife, fishing resources, water quality, and water quantity), the interviewees needed to organize numerically from least effect to highest effect using a scale of 0 to 5. The options were related to threats, consequences of loss, and solutions to the loss of forests, wetlands, wildlife, fishing resources, water quality, and water quantity. The options for each resource were defined in consensus with AMUPROLAGO, based on their local experience in the region as comanagers of the watershed. However, interviewees could add other options they deemed necessary.

**Table 1:** Descriptive statistics of the sample.

Attribute	Mean	Min	Max	Std
**Population**	568.30	109.00	1490.00	438.46
**Distance to lake**	1218.33	27.32	2626.94	878.56
**Slope**	8.26	1.48	20.96	6.01
**% forest**	20.07	5.64	57.68	14.4
**% pasture/crops**	42.02	19.19	72.29	17.47
**% coffee plantation**	11.85	0.00	54.20	18.17
**Time living in the area**	23.75	0.00	68.00	14.75
**Attribute Class**	Number	Frequency		
**Age**				
**15–30**	56	25%		
**31–45**	108	48.2%		
**46–60**	39	17.4%		
**> = 60**	21	9.4%		
**Organizations**	Total-24			
**Producers**	12	54%		
**Government**	6	27%		
**Non-governmental organizations (NGOs)**	6	27%		
**Community members**	Total-200			
**Conservation**	1	0.45		
**Production/service**	85	42.4		
**Tourism**	27	13.4		
**Community service**	63	31.3		
**Unemployed**	4	1.8		

The information provided in the surveys was transferred into a database, and some variables were classified, tabulated, and organized for statistical analysis (See supplementary files). For example, the answers for the work variable were classified into a categorical variable of six levels based on two grouping categories (organizations and community members): tourism, production/service, community services, unemployment, conservation, government, NGO, and producers.

The answers to the three component questions in the survey were numerical responses, where interviewees classified the proposed options with a number between 0–5 (Tables 3 to 7). Where 0 represented that these options had no effect on the resource in question, 1 represented a low relevance value for a given option, and 5 represented the highest relevance value. We did our best to mitigate confirmation bias by clarifying the survey structure, the purpose of the project research, and its implementation to each respondent. Nevertheless, there could still be some confirmation bias that is difficult to avoid, like in any social science research.

For the three component questions, answers were tabulated and organized to recognize which option was considered the most relevant for each component within each resource. To assess the leading threats, impacts, and solutions perceived by the communities associated with the loss of each specific natural resource, the percentage of each numerical value (0–5) was used. These top choices were considered as the dependent variable for our statistical analyses. Ordinal logistic regression analysis in R software was used to determine which demographic, land use, or opinion factors could affect the communities’ decisions. Statistical analysis was based on the type of numerical data observed for our response variable. The dependent variables used were the options considered the most relevant (highest rate) for threats, consequences of loss, and solutions for each resource (forests, wetlands, wildlife, fishing resources, water quality, and water quantity). We use the Akaike Information Criterion (AIC) as a test for model fitness [62] and evaluate the significance levels of coefficients with a t-test at p≤ 0.05. We tested the ordinal regression for proportional odds (assumption of parallel lines).

The independent demographic variables (Table 1) used in the analyses were community population size, estimated age, time living in the area, and job. The opinion-based independent variables used in the analysis were the perception of the importance of tourism, the magnitude of threat (variation in the value of relevance of the most important threat selected for each resource), the magnitude of impact (variation in the value of relevance of the most important option consequence of loss chosen for each resource), and leading solutions to mitigate the impacts.

The land uses independent variables (Table 1) used in the analyses were distance to the lake from each community, percentages of land uses (forest, pasture/crops, and coffee plantations) within a 1km buffer zone around the community, and average slope within a 1km buffer zone around each community. Landscape variables were obtained through GIS analyses of the 2018 Land use layer and an Elevation layer for Honduras. The land use data were obtained from the Institute for the Conservation and Development of Forests, Protected Areas, and Wildlife in Honduras (*Instituto de Conservación y Desarrollo Forestal Áreas Protegidas y Vida Silvestre*, ICF). The elevation and slope layers were obtained from the GDEM (global digital elevation map) downloaded from a NASA webpage (https://reverb.echo.nasa.gov). All maps were projected at WGS_1984_UlikeTM_Zone 16N and used the extent and cell size of the DEM for any raster analysis (30 m resolution).

## Results

Descriptive statistics of variables used in the analysis are presented in Table 1. In addition, various demographic, land use, or opinion factors were used as independent variables in the analyses (Tables 2–7). The leading threat perceived by local communities for forests is local wood consumption, while economic activities pose a primary threat to wetlands. Loss in terrestrial and aquatic habitats was identified as a threat to wildlife and fisheries resources. The loss of forests and wetlands was considered a major threat to water resources in the watershed. Consequences of forest and wetland loss are the loss of water resources, while impacts of fishery loss are attributed to loss of jobs and income. Wildlife loss was perceived to impact other related species in general, and the consequence of loss in water resources was attributed to loss in public health. Reforestation was a primary solution suggested to protect forests and wetlands and to improve water quantity. For further analysis of TCS components, the results of two models are presented: the complete model that included all variables in the analyses and the AIC-selected model.

**Table 2:** Demographic and landscape characteristics influencing perceptions of leading threats to natural resources*.

Variables	Forest		Wetlands		Wildlife		Fisheries		Water quality		Water quantity	
Leading Threat	Local wood consumption		Economic activities		Loss of habitat		Loss of habitat		Loss of forests and wetlands		Loss of forests and wetlands	
Model Type	*Complete model*	*AIC Selected*	*Complete model*	*AIC Selected*	*Complete model*	*AIC Selected*	*Complete model*	*AIC Selected*	*Complete model*	*AIC Selected*	*Complete model*	*AIC Selected*
AIC	692.483	691.935	660.278	650.072	604.900	584.687	734.542	734.477	628.669	623.804	596.680	587.748
Classification error	0.621	0.603	0.649	0.681	0.442	0.442	0.693	0.697	0.478	0.478	0.487	0.4777
Population											**-0.001**	**-0.001**
Distance to lake					**0.001**							
Forests %					**-0.030**							**-0.024**
Pasture/crops %					**-0.039**	**-0.014**						
Coffee plantation %		-0.013		**-0.018**	**-0.031**		***-0*.*013***	**-0.017**				
Slope		**-0.048**										**0.0442**
Time living in area	**-0.02**	**-0.029**	**0.024**				***0*.*016***					
*Work*												
unemployed		Control	Control		Control		Control		Control		Control	
community services		0.020	0.544		0.072		-1.322		0.309		-0.503	
conservation		0.261	0.032		**0.257**		**15.476**		***-2*.*901***		-1.931	
government		**-2.601**	1.865		**-1.929**		-1.484		-1.354		***-1*.*419***	
NGO		***-2*.*262***	**2.557**		**-0.558**		-0.309		0.701		-0.818	
producers		-1.751	1.083		-0.373		-0.893		0.8307		0.723	
production/service		-0.582	0.910		**-0.589**		-1.984		0.147		-0.553	
tourism		-0.491	0.598		0.114		-1.279		0.111		**-1.040**	
*Estimated age*												
>30	Control	Control	Control									
31–45 yrs.	-0.031	0.230	-0.481									
46–60 yrs.	-0.401	0.075	**-0.999**									
≥60 yrs.	**1.131**	**1.565**	-0.864									

*Coefficients in bold represent variables that are significant by a 95% confidence or higher.

Coefficients in bold and underlined represent variable that are significant by a 90–94% confidence.

**Table 3:** Opinions influencing perceptions of leading threats to natural resources*.

Variables	Forest		Wetlands		Wildlife		Fisheries		Water quality		Water quantity	
Leading Threat	Local wood consumption		Economic activities		Loss of habitat		Los of habitat		Loss of forests and wetlands		Loss of forests and wetlands	
Model Type	*Complete model*	*AIC Selected*	*Complete model*	*AIC Selected*	*Complete model*	*AIC Selected*	*Complete model*	*AIC Selected*	*Complete model*	*AIC Selected*	*Complete model*	*AIC Selected*
AIC	692.483	691.935	660.278	650.072	604.900	584.687	734.542	734.477	628.669	623.804	596.680	587.748
Classification error	0.621	0.603	0.649	0.681	0.442	0.442	0.693	0.697	0.478	0.478	0.487	0.4777
*Tourism Importance*												
No					Control		Control	Control				Control
yes and no					**0.383**		1.082	1.351				***-1*.*676***
yes					-0.250		**-1.050**	***-0*.*739***				-0.705
Magnitude of consequences (scale 0–5, control = 0)			1.189 **2.463** **2.281** **1.502** **1.913**	1.026 **2.223** **2.055** **1.328** **1.939**	**-1.087** 0.113 -0.183 -0.083 0.0190				**2.849** **2.296** **2.568** ***1*.*813*** **2.158**	2.014 **2.506** **2.659** ***1*.*835*** **2.156**	0.630 **1.642** 0.240 0.549 0.763	0.194 **1.845** 0.178 0.604 0.797

*Coefficients in bold represent variables that are significant by a 95% confidence or higher.

Coefficients in bold and underlined represent variable that are significant by a 90–94% confidence

**Table 4:** Demographic and landscape characteristics influencing perceptions of leading consequences experienced by the loss of natural resources*.

Variables	Forest		Wetlands		Wildlife		Fisheries		Water quality		Water quantity	
consequences experienced by the loss of resource	Loss of Water resources		Loss of Water resources		Loss of other species		Loss of jobs and income		Loss of public health		Loss of public health	
Model Type	*Complete model*	*AIC Selected*	*Complete model*	*AIC Selected*	*Complete model*	*AIC Selected*	*Complete model*	*AIC Selected*	*Complete model*	*AIC Selected*	*Complete model*	*AIC Selected*
AIC	584.026	582.188	598.626	586.830	665.285	661.881	744.311	735.126	377.947	374.997	629.454	None
Classification error	0.527	0.536	0.601	0.601	0.522	0.527	0.647	0.651	0.205	0.205	0.464	
Distance to lake	**0.001**		**-0.001**				4.0*10 ^**−4**^					
Forests %	**-0.041**		**0.044**	**-0.019**				**-0.023**				
Pasture/crops %	**-0.028**		**0.049**				**0.017**					
Coffee plantation %	**-0.033**		**0.040**					**-0.022**				
Slope	**-0.058**	**-0.049**					**0.057**	***0*.*039***				
Time living in area	**0.024**	***0*.*017***										
*Work*												
unemployed	Control		Control		Control		Control		Control		Control	
community services	**-1.205**		**-0.795**		0.668		**-1.759**		**-16.043**		-0.474	
conservation	**-1.309**		**-1.922**		-1.310		**-2.386**		**0.161**		6.860	
government	**-1.090**		**2.476**		1.597		***-1*.*221***		**-17.87**		***-2*.*020***	
NGO	**-3.227**		**2.945**		1.675		-0.675		**-0.078**		-0.806	
producers	**-1.238**		**2.515**		**2.219**		***-0*.*840***		**-16.736**		-0.898	
production/service	**-1.589**		**-0.801**		0.982		**-1.914**		**-15.926**		-0.146	
tourism	***-0*.*498***		**-1.451**		1.089		**-1.385**		**-15.502**		0.171	
*Estimated age*												
>30					Control	Control						
31–45 yrs.					-0.312	-0.214						
46–60 yrs.					-0.669	-0.561						
≥60 yrs.					**-1.327**	**-1.164**						

*Coefficients in bold represent variables that are significant by a 95% confidence or higher.

Coefficients in bold and underlined represent variable that are significant by a 90–94% confidence.

**Table 5:** Opinions influencing perceptions of leading consequences experienced by the loss of natural resources.

Variables	Forest		Wetlands		Wildlife		Fisheries		Water quality		Water quantity	
Impact experienced by the loss of resource	Loss of Water resources		Loss of Water resources		Loss of other species		Loss of jobs and income		Loss of public health		Loss of public health	
Model Type	*Complete model*	*AIC Selected*	*Complete model*	*AIC Selected*	*Complete model*	*AIC Selected*	*Complete model*	*AIC Selected*	*Complete model*	*AIC Selected*	*Complete model*	*AIC Selected*
AIC	584.026	582.188	598.626	586.830	665.285	661.881	744.311	735.126	377.947	374.997	629.454	None
Classification error	0.527	0.536	0.601	0.601	0.522	0.527	0.647	0.651	0.205	0.205	0.464	
*Tourism Important*												
No			Control				Control		Control	Control		
yes and no			***-0*.*526***				0.210		**15.532**	**14.215**		
yes			0.567				***0*.*650***		-0.422	-0.548		

*Coefficients in bold represent variables that are significant by a 95% confidence or higher. Coefficients in bold and underlined represent variable that are significant by a 90–94% confidence.

**Table 6:** Demographic and landscape characteristics influencing perceptions of the leading solutions to loss of natural resources*.

Variables	Forest		Wetlands		Wildlife		Fisheries		Water quality		Water quantity	
Leading solution	Reforestation		Reforestation		Law enforcement		Law enforcement		Agroforestry		Reforestation	
Model Type	*Complete model*	*AIC Selected*	*Complete model*	*AIC Selected*	*Complete model*	*AIC Selected*	*Complete model*	*AIC Selected*	*Complete model*	*AIC Selected*	*Complete model*	*AIC Selected*
AIC	629.127	628.254	641.637	636.415	806.911	790.655	717.646	704.188	792.112	774.527	642.024	635.315
Classification error	0.505	0.5	0.654	0.649	0.692	0.701	0.601	0.596	0.683	0.683	0.486	0.487
population	**0.001**											
distance to lake					**-0.001**		**-0.001**		**0.001**			
Forests %	**0.051**	**0.032**			**0.060**		**0.086**		**-0.053**			
pasture/crops %	**0.029**	**0.019**			**0.035**	**-0.014**	**0.064**		**-0.036**		**0.014**	**0.017**
coffee plantation %	**0.029**	**0.025**			**0.031**		**0.060**		**-0.044**			**0.023**
slope					***0*.*041***							
*Work*												
unemployed	Control		Control		Control		Control		Control			
community services	-0.238		-1.371		**0.799**		**0.631**		**1.784**			
conservation	**-2.837**		**-3.908**		**0.400**		**0.573**		**1.644**			
government	0.679		***-2*.*381***		**3.960**		**4.963**		0.053			
NGO	-0.813		***-2*.*702***		**3.778**		**4.568**		-0.455			
producers	**1.516**		-1.535		**4.434**		**4.892**		**-0.216**			
production/servic	-0.716		-1.658		**1.121**		**0.813**		**1.912**			
e tourism	-0.893		-1.288		**0.781**		**0.744**		**1.050**			
*estimated age*												
>30	Control	`Control					Control	Control			Control	Control
31–45 yrs.	**0.889**	**0.721**					***0*.*511***	0.498			**0.760**	**0.854**
46–60 yrs.	**1.213**	**1.001**					**0.957**	***0*.*751***			**0.988**	**1.137**
≥60 yrs.	-0.059	-0.180					**1.758**	**1.773**			0.325	0.390

*Coefficients in bold represent variables that are significant by a 95% confidence or higher.

Coefficients in bold and underlined represent variable that are significant by a 90–94% confidence

**Table 7:** Opinions influencing perceptions of the leading solutions to loss of natural resources*.

Variables	Forest		Wetlands		Wildlife		Fisheries		Water quality		Water quantity	
Leading solution	Reforestation		Reforestation		Law enforcement		Law enforcement		Agroforestry		Reforestation	
Model Type	*Complete model*	*AIC Selected*	*Complete model*	*AIC Selected*	*Complete model*	*AIC Selected*	*Complete model*	*AIC Selected*	*Complete model*	*AIC Selected*	*Complete model*	*AIC Selected*
AIC	629.127	628.254	641.637	636.415	806.911	790.655	717.646	704.188	792.112	774.527	642.024	635.315
Classification error	0.505	0.5	0.654	0.649	0.692	0.701	0.601	0.596	0.683	0.683	0.486	0.487
*Tourism*												
No			Control		Control		Control		Control			
yes and no			-0.117		**-0.568**		**0.839**		**-0.840**			
yes			***1*.*003***		-0.471		0.092		-0.211			
Magnitude of consequences (scale 0–5, control = 0)			**2.095** **2.823** **3.073** **3.081** **2.623**	**1.782** **2.415** **2.602** **2.952** **2.323**	**-1.284** **-1.075** **-0.898** **-1.499** **-1.168**	-1.003 ***-1*.*117*** ***-0*.*846*** **-1.445** **-1.086**	**-0.143** -0.622 -0.519 -0.259 ***0*.*448***		**3.559** **3.332** **3.115** **2.865** **3.572**	**3.707** **3.246** **3.177** **3.052** **3.500**		
Magnitude of threat (scale 0–5, control = 0)	0.348 -0.170 **1.609** 0.784 0.609	0.171 -0.257 **1.505** 0.839 0.5049			0.067 **-1.085** -0.496 -0.271 **-0.761**				0.397 **0.861** **1.461** **0.746** **0.867**		**3.127** **4.897** **4.871** **4.715** **5.157**	**3.478** **5.341** **5.064** **5.070** **5.474**

*Coefficients in bold represent variables that are significant by a 95% confidence or higher.

Coefficients in bold and underlined represent variables that are significant by a 90–94% confidence

### Threats

The analyses for leading threats (Tables 2 and 3) show that several variables could affect the interviewees’ choices. The factors with a high level of significance for the leading threats were population size, distance to the lake, landcover percentages (forests, pasture/crops, coffee plantations), slope, amount of time living in the area, type of work, estimated age, the importance of tourism and the magnitude of consequences provided for that resource. However, the magnitude of consequences for that resource, type of work, amount of time living in the area, and landcover percentages were more commonly found significant throughout many of the resources studied.

The variables used to analyze the perception of leading threats to natural resources were divided into two categories: demographic and landscape (Table 2) and opinions (Table 3). In the demographic and landscape category (Table 2), forests had the highest quantity of relevant variables. The AIC selected model showed slope (-0.048), time living in the area (-0.029), government work (-2.601), and age greater than 60 years. (1.565) where the most significant variables are the leading perceived threat to forests. The coffee plantation % was the most significant variable for wetlands (-0.018) and fisheries (-0.017). Pasture /crop % (-0.014) was the most significant for wildlife, while population (-0.001) and forest percentage (-0.024) were the most significant for water quantity. There were no significant variables for water quality in the demographic and landscape category. In the opinions category (Table 3), considering tourism important was significant for fisheries (-0.739) and water quality (-1.676). The magnitude of impacts was relevant for wetlands, water quality, and water quantity.

### Consequences

The analyses for leading consequences (Tables 4 and 5) show that several variables could affect our interviewees’ choices. The factors that showed a high significance level for the leading consequences were distance to the lake, landcover percentages (forests, pasture/crops, coffee plantations), slope, amount of time living in the area, type of work, estimated age, and importance of tourism. The job type, slope, the importance of tourism, and landcover percentages were found throughout many of the resources.

The variables used to analyze the perception of leading consequences due to the loss of natural resources were divided into two categories: demographic and landscape (Table 4) and opinions (Table 5). In the demographic and landscape category (Table 4), fisheries had the highest quantity of relevant variables. The AIC selected model showed that forest % (-0.023) and coffee plantation % (-0.022) were the most significant variables for the leading perceived consequences of fisheries loss. Forest percentage (-0.019) was the most significant for the perceived consequence of wetlands loss, while slope (-0.049) was the most significant for the perceived consequence of forest loss. There were no significant variables for wildlife, water quality, and water quantity in the demographic and landscape category. In the opinions category (Table 5), considering tourism important was significant for water quality (14.215).

### Solutions

The analyses for leading solutions (Tables 6 and 7) show that several variables could affect our interviewees’ choices. The factors with a high level of significance for the leading solutions were population size, distance to the lake, landcover percentages (forests, pasture/crops, coffee plantations), slope, type of work, estimated age, the importance of tourism, the magnitude of threat provided for that resource and the magnitude of consequences provided for that resource. Where landcover percentages, type of work, estimated age, the importance of tourism, the magnitude of impact, and the magnitude of threat were the most found in a general way throughout many of the resources.

The variables used to analyze the perception of leading solutions for the loss of natural resources were divided into two categories: demographic and landscape (Table 6) and opinions (Table 7). In the demographic and landscape category (Table 6), forests had the highest quantity of relevant variables. The AIC selected model showed forest % (-0.032), pasture/crop % (0.019), coffee plantation % (0.025), and age ranges of 31–45 yrs. (0.721) and 46–60 yrs. (1.001) where the most significant variables are the leading perceived solutions to forest loss. Pasture/crop percentage (-0.014) was the most significant for the leading perceived solution of wildlife loss. Age greater than 60 years. (1.773) was the most significant for the leading perceived solution of fisheries loss. The most significant variables for the leading perceived solution to water quantity were losses were pasture/crop % (0.017), coffee plantation % (0.023), and age ranges of 31–45 yrs. (0.854) and 46–60 yrs. (1.137). No significant wetland and water quality variables were in the demographic and landscape category. In the opinions category (Table 7), the magnitude of the consequences variable was relevant for the leading perceived solutions to the loss of wildlife, wetlands, and water quality. On the other hand, the magnitude of threats and variability was relevant for the leading perceived solutions to the loss of forests and water quantity. Management strategies to improve watershed resilience can use information on the role of factors in mitigating threats to resources.

## Discussion

This study uses a TCS framework to evaluate the local context that drives the perceptions and values of watershed stakeholders regarding several common pool resources in Lake Yojoa. A survey of local communities in the watersheds showed that the value of resources varied as a factor in the communities’ interactions with them. Some communities, for example, could not identify with wetlands, as they were situated higher up the mountains and farther away from the lake. This observation is consistent with past research on perception towards wetlands as linked to biophysical characteristics of the landscape [63]. Likewise, those with no constraints in accessing resources could not identify solutions to possible threats or impacts. However, those with difficulty accessing these resources were able to identify the threats and effects of the loss of these resources. This result is consistent with observation on the role of the perceived seriousness of the problem as a necessary condition for conservation solutions [64]. These variations motivated the development of a conceptual framework based on TCS to assess factors driving stakeholder-resource interactions influencing the resilience of local commons. The analyses found six significant factors across the TCS components. These six factors are distance to the lake, landcover percentages (forests, pasture/crops, coffee plantations), slope, type of work, estimated age, and importance of tourism. Other studies on resource values have found similar results. For example, Yang *et al*. [39] used a survey to assess the local communities’ perception of forest-related values and their attitude toward managing the Bulong Nature Reserve (BNR) in Yunnan, China. The study showed that many social factors affected the observed variation in perception and attitudes, including age, gender, education, and distance from the reserve. Likewise, Oteros-Rozas *et al*. [33] studied residents and tourists of the Conquense Drove Road area in Spain to understand the importance of 34 ecosystem services associated with this region. Based on these interviews and their responses, the value of ecosystem services varied because of social differences such as age, place of origin, and gender.

The types of work seem to have a high effect on defining the threats, consequences, and solutions analyses. In the case of the threats analyses, types of work showed this high effect when related to government, NGOs, or conservation. It indicates that a more in-depth knowledge of the area could provide a stronger sense of the threats and resource threats of the analyses of the consequences, and types of work showed this high effect when the work was related to products and services. This observation could indicate that higher dependence on resources, as expected with these work categories, could provide a stronger sense of the impacts of the loss of resources. In the case of the solutions analyses, types of work showed this high effect when the work was related to government, NGOs, conservation, and production. This result could indicate that a more in-depth knowledge of the area and dependence on resources, as expected with these work categories, could provide a stronger sense of the solutions to the loss of resources and improve the resilience of local commons. Differences in perceptions of ecosystem services among stakeholder types were also documented in rural landscapes [65].

The magnitude of the consequences perceived by the interviewees was another significant influencing factor in the threat analyses. This result could mean that how the loss of natural resources affects them personally could influence their perception of what is threatening these natural resources. For example, the impacts of droughts on water availability and food were perceived by small subsistence communities in the Yucatan peninsula of Mexico to change traditional cropping systems [66]. The importance of tourism, as perceived by communities, was another significant influencing factor in analyzing the consequences. Communities are also concerned that the loss of natural resources, for example, water quality, could influence tourism growth in the area [67]. Communities perceiving higher economic crisis and place attachment were found to support ecotourism [68]. Low local involvement in ecotourism is attributed to a lack of a benefit-sharing mechanism [69]. The magnitude of the threats and consequences perceived by the interviews were two other factors of significant influence in the solutions analyses. The perception of how the loss of natural resources affects them personally and what is causing these losses would influence the perception of the best solutions. For example, climate change impacts on water resources are perceived to affect the livelihood of local communities who use adaptation strategies based on traditional knowledge to enhance food security [70].

To improve the resilience of local commons, the effects of social factors on perceptions need to be recognized in managing watershed commons. Identifying potential threats through community participation is critical to reducing the risk of loss in ecosystem services. This recommendation will involve making changes to local practices and institutional mechanisms. There is a need to understand the potential impact of resource loss on communities, assess the resource’s state, and evaluate any local or regional variations. Unbalanced community participation can affect conservation and ecosystem management effectiveness in achieving the resilience of commons. Encouraging local communities to participate in finding solutions can result in community support and ongoing community involvement, which will increase the success of conservation efforts towards resilience of local commons.

## Conclusions

To make environmental conservation programs and strategies more effective in enhancing the resilience of local commons, understanding what motivates people’s perceptions of the local commons they depend upon is crucial. Community members have different priorities and perspectives and need careful assessment of factors influencing them. Therefore, it is critical to take a holistic approach when examining the factors influencing people’s perceptions, including the biotic, abiotic, and anthropogenic factors. These factors constantly interact and impact people’s well-being and should also be considered.

This study identifies several demographic, landscape, and opinion factors that impact the perception of leading threats and solutions to the loss of common pool resources in the Lake Yojoa watershed in Honduras. This information will serve as a valuable baseline for conservation and governmental organizations as they work on watershed management plans and conservation strategies. In addition, the TCS approach could be used to study watershed commons for community planning and management by considering the perspectives of various stakeholders and community members.

The summary of the threats, consequences of loss, and proposed solutions to the loss of common pool resources is an excellent baseline information for creating conservation and use strategies for common pool resources (Table 8). Understanding what the communities identify as threats to the commons can allow watershed communities to mitigate and adapt to threats through incentive mechanisms and management for enhancing resilience. The perceived impacts can be a basis for community-based solutions that involve local knowledge that promotes conservation for sustainable outcomes. The community’s perceptions of threats, consequences, and solutions are the first step in a community-based approach to managing watershed commons. Landscape factors like distance to the lake, land cover, and slope played a role in influencing TCS perception. They could help understand the implications of changing landscape and social factors on perceptions. A cooperative effort in identifying threats, consequences, and solutions can be used as a framework for public participation and management in various watershed commons. Unbalanced community participation in management can impair conservation and sustainable ecosystem use effectiveness. The general assessment of TCS can also be used to study differences among households’ dependence on natural systems and increase support for better local participation in conservation programs, especially in regions developing efforts towards ecotourism, wetland conservation, forest restoration, and water quality protection.

**Table 8:** Leading threats, impacts, and solutions perceived by the communities due to the loss of each specific natural resource.

	Forests	Wetlands
Threat to this resource	Local wood consumption	Economic Activities
Consequences due to the loss of this resource	Loss of water resources (47.8%)	Loss of wildlife (32.6%)
	Loss of Health (31.7%)	Loss of water resources (20.5%)
		Loss of Health (20.1%)
Solutions to the loss of this resource	Reforestation (45.5%)	Reforestation (25.4%)
		Stronger support for law reinforcement (21.4%)
	Wildlife	Fisheries
Threat to this resource	Loss of habitat	Loss of habitat
Consequences due to the loss of this resource	Loss of wildlife species (55.8%)	Loss of income and employment (32.1%)
	Loss of Health (20.5%)	Loss of Food resources (28.1%)
Solutions to the loss of this resource	Stronger support for law reinforcement (28.6%)	Stronger support for law reinforcement (39.3%)
	Reforestation (27.2%)	
	Water Quality	Water Quantity
Threat to this resource	Loss of natural resources	Loss of natural resources
Consequences due to the loss of this resource	Loss of Health (79.5%)	Loss of health (53.6%)
		Loss of water availability (22.3%)
Solutions to the loss of this resource	Agroforestry (29.9%)	Reforestation (49.1%)
	Reforestation (24.1%)	

Further interdisciplinary research is vital for better understanding the dynamics of TCS and how communities perceive these at a watershed scale. Future research can focus on factors like uncertainty, large projects, and policies that may influence the attitudes and values observed at the community level. While this study identified threats, consequences, and solutions for each resource across all communities, decision-makers would benefit from conducting a similar analysis in each community and larger regions in assessing baseline states and paths to enhance the resilience of local commons. This approach would provide a more comprehensive framework for understanding emerging watershed commons threats and local knowledge towards sustainable solutions.

## Supporting information

S1 File (DOCX)

S1 Questionnaire Measure of communities’ value on ecosystem health. (DOCX)

S1 Data (CSV)
