# Correction: A sustainability framework based on threats, consequences, and solutions (TCS) for managing watershed commons

**DOI:** 10.1371/journal.pone.0346555

**Published:** 2026-04-06

**Authors:** Ana Lorena Quiñónez Camarillo, Timothy O. Randhir

The initials of the first author are indexed incorrectly in PubMed. The correct initials are: Quiñónez Camarillo, AL.

The correct citation is: Quiñónez Camarillo, AL, Randhir TO (2023) A sustainability framework based on threats, consequences, and solutions (TCS) for managing watershed commons. PLoS ONE 18(12): e0295228. https://doi.org/10.1371/journal.pone.0295228
