# Supplementary material for: A sustainability framework based on threats, consequences, and solutions (TCS) for managing watershed commons
Source: PLoS One. 2023 Dec 6;18(12):e0295228. doi: 10.1371/journal.pone.0295228 (PMC10699595; doi:10.1371/journal.pone.0295228)
Supplement: S1 File — (DOCX) [file pone.0295228.s001.docx]

Metadata for:

**A sustainability framework based on threats, consequences, and solutions (TCS) for managing watershed commons.**

The surveys were applied between the months of June-July of 2019.

We have 28 variables used in the analyses of the data. All variables are described below.

**Population** - number of individuals in each community surveyed

**dist.lake** – distance to lake from each community

**Slope** – mean slope for each community

**Forests** – forest % coverage in a 1 km radius around each community

**Pasture/crops**- Pasture/crops % coverage in a 1 km radius around each community

**Cofee.plant** – Coffee plantation % coverage in a 1 km radius around each community

**tlia** - years living in the area

**estage** - estimated age ranges

**Work** - work categories under which all work options provided by interviews have been grouped into

*Organizations****:***

Producers

Government

Non-governmental organizations (NGO)

*Community members:*

Conservation

Production/service

Tourism

Community service

Unemployed

**TourImp**: perception of the importance of tourism, yes or no question

The las 18 variables represent the variation in choices for the most important threat to, consequence of the loss of and solutions to the loss of each specific resource being analyzed. The first one or two letters of the variable name are referring to the specific recourse being considered (e.g. F for forest, WL for wetland, WR for wildlife resources, FR for fisheries resources. QL for water quality, and QN for water quantity resource. The first letters(s) were followed by a T, A, or S to identify the variable as a threat, consequence (represented by the A for affected area) or solution. The last letters represent the specific threat, consequence, or solution of each resource.

Forest variables

**FTLC**

**FAWR**

**FSR**

Wetland variables

**WLTB**

**WLALW**

**WLSR**

Wildlife resources variable

**WRTLNR**

**WRALW**

**WRSL**

Fisheries resource variable

**FRTLNR**

**FRAJ**

**FRSL**

Water Quality variables

**QLTLNR**

**QLAH**

**QSAF**

Water Quantity variables

**QNTLNR**

**QNAH**

**QNSR**
