# Supplementary material for: A sustainability framework based on threats, consequences, and solutions (TCS) for managing watershed commons
Source: PLoS One. 2023 Dec 6;18(12):e0295228. doi: 10.1371/journal.pone.0295228 (PMC10699595; doi:10.1371/journal.pone.0295228)
Supplement: S1 Questionnaire — (DOCX) [file pone.0295228.s002.docx]

**Measure of communities’ value on ecosystem health**

**Proposed by: Ana Quinonez**

My name is Ana Quiñónez and I am a student at the University of Massachusetts doing my research in conservation efforts in tropical watersheds. I am surveying people who live and work in the Lake Yojoa watershed. This survey is designed to measure the value that communities’ place on their natural resources. WE NEED YOUR IMPUT!! By learning how stakeholders feel towards their surrounding natural resources and their health, we are able to use this information to develop conservation actions and recommendations that apply to this area in particular.

Every reasonable effort has been made to maintain the confidentiality of the data, and it will be released only in a summary form and for statistical analyses, in which no individual answer can be identified. This survey is voluntary and should take about 10 minutes to complete. If you are consenting to this survey, please help us fill in the information requested below and answer the questions located in the next pages. If you are not willing to take the survey, please stop here. Thank you for taking the time to participate in this survey and contributing to this important research.

Sincerely,

Ana Quiñónez Dr. Timothy Randhir

PHD Student Professor

Dept. Environmental Conservation Dept. Environmental Conservation

University of Massachusetts University of Massachusetts

**Questionnaire no.: ________________________ Date of completion: ________________________**

**Community: __________________________________________________________________________**

**Estimated age of interviewee (do not ask, just circle your estimate)**

≤ 15 yrs. 15 – 30 yrs. 30 – 45 yrs. 45 – 60 yrs. $\geq$60 yrs

**How long have you been living in the area?**

**What do you do for a living?**

**Measure of communities’ value on ecosystem health**

1. a. What are the threats that affect local forests and wetlands? (Number your choices between 0-5, O being no impact, 1 being the one that represents the least threat and 5 the most threat)

| *Threat* | *Forest* | *Wetland* |
| --- | --- | --- |
| Agricultural practices |  |  |
| Residential development |  |  |
| Economics activities (businesses, ecotourism, energy production, mines, aquaculture) |  |  |
| Local consumption (firewood, home, cattle) |  |  |
| Ecotourism |  |  |
| other |  |  |

b. How does the loss of forest and wetlands affect you? (Number your choices between 0-5, O being an area not affected, 1 being the area least affected and 5 the area most affected).

| *Areas affected by loss* | *Loss of Forest* | *Loss of Wetland* |
| --- | --- | --- |
| Health |  |  |
| Income and employment |  |  |
| Economics activities (businesses, ecotourism) |  |  |
| Water Resources |  |  |
| Loss of Wildlife |  |  |
| other |  |  |

c. How can this be changed? (Number your choices between 0-5, O being an option not considered a solution, 1 being the one that represents the least helpful solution and 5 the most helpful solution).

| *Solutions* | *Forest* | *Wetland* |
| --- | --- | --- |
| Agroforestry |  |  |
| Stronger support for law reinforcement (protection and loss regulation) |  |  |
| Special community harvest areas |  |  |
| Forestry and wetland education programs |  |  |
| Ecotourism |  |  |
| Reforestation |  |  |
| Conservation zoning |  |  |
| other |  |  |

1. a. What are the threats that affect local wildlife presence (bird, otter, etc.) and fish stock? (Number your choices between 0-5, O being no impact, 1 being the one that represents the least threat and 5 the most threat)

| *Threat* | *Wildlife* | *Fish stock* |
| --- | --- | --- |
| Agricultural practices |  |  |
| Residential development |  |  |
| Economics activities (businesses, ecotourism, energy production, mines, aquaculture) |  |  |
| Local consumption (fishing, hunting) |  |  |
| Ecotourism |  |  |
| Loss of natural areas (forests, wetlands, rivers) |  |  |
| other |  |  |

b. How does the loss of local wildlife presence (bird, otter, etc.) and fish stock affect you? (Number your choices between 0-5, O being an area not affected, 1 being the area least affected and 5 the area most affected).

| *Areas affected by loss* | *Loss of Wildlife* | *Loss of Fish Stock* |
| --- | --- | --- |
| Health |  |  |
| Income and employment |  |  |
| Economics activities (businesses, ecotourism) |  |  |
| Food Resources |  |  |
| Loss of other wildlife species |  |  |
| other |  |  |

c. How can this be changed? (Number your choices between 0-5, O being an option not considered a solution, 1 being the one that represents the least helpful solution and 5 the most helpful solution).

| *Solutions* | *Wildlife* | *Fish Stock* |
| --- | --- | --- |
| Agroforestry |  |  |
| Stronger support for law reinforcement (protection and loss regulation) |  |  |
| Special community harvest areas |  |  |
| Forestry, wetland, and wildlife education programs |  |  |
| Ecotourism |  |  |
| Reforestation |  |  |
| Conservation zoning |  |  |
| other |  |  |

1. a. What are the threats that affect local water quality and quantity? (Number your choices between 0-5, O being no impact, 1 being the one that represents the least threat and 5 the most threat)

| *Threat* | *Water Quality* | *Water Quantity* |
| --- | --- | --- |
| Land use (urban development, agriculture) |  |  |
| Lack of used water treatment facilities |  |  |
| Economics activities (businesses, ecotourism, energy production, mines, aquaculture) |  |  |
| Unsustainable water withdrawals |  |  |
| Loss of natural areas (forests, wetlands, rivers) |  |  |
| other |  |  |

b. How does the loss of local water quality and quantity affect you? (Number your choices between 0-5, O being an area not affected, 1 being the area least affected and 5 the area most affected).

| *Areas affected by loss* | *Loss of Water Quality* | *Loss of Water Quantity* |
| --- | --- | --- |
| Health |  |  |
| Loss of natural resources (forests, wetlands, wildlife) |  |  |
| Economics activities (businesses, ecotourism) |  |  |
| Food Resources |  |  |
| Water availability |  |  |
| other |  |  |

c. How can this be changed? (Number your choices between 0-5, O being an option not considered a solution, 1 being the one that represents the least helpful solution and 5 the most helpful solution).

| *Solutions* | *Water Quality* | *Water Quantity* |
| --- | --- | --- |
| Agroforestry, less use of chemicals |  |  |
| Stronger support for law reinforcement (protection and loss regulation) |  |  |
| Better drainage and treatment systems for used waters (gray and sewage) |  |  |
| Forestry, wetland, and adequate water use education programs |  |  |
| Reforestation |  |  |
| Conservation zoning |  |  |
| other |  |  |

1. a. Is tourism important for the Lake Yojoa Watershed? Why?

b. What are the threats to tourism in the Lake Yojoa Watershed?
